# Dynamic Changes in Plasma MicroRNAs Have Potential Predictive Values in Monitoring Recurrence and Metastasis of Nasopharyngeal Carcinoma

**DOI:** 10.1155/2018/7329195

**Published:** 2018-01-18

**Authors:** Xia Xu, Juan Lu, Fan Wang, Xiong Liu, Xiaohong Peng, Bolong Yu, Feipeng Zhao, Xiangping Li

**Affiliations:** ^1^Department of Otolaryngology-Head and Neck Surgery, Guangzhou General Hospital of People's Liberation Army of China, Guangzhou, Guangdong 510010, China; ^2^Department of Otolaryngology-Head and Neck Surgery, Nanfang Hospital, Southern Medical University, Guangzhou, Guangdong 510515, China

## Abstract

Although circulating microRNAs (miRNAs) have already proven to be useful as diagnostic and prognostic biomarkers in nasopharyngeal carcinoma (NPC), the potential of these molecules to monitor patients over time has been less explored. This study aimed to analyze dynamic changes in plasma miRNAs before and after treatment and explore their clinical significance in monitoring recurrence and metastasis of NPC. Candidate miRNAs were screened by microarray analysis and validated by real-time quantitative polymerase chain reaction (RT-qPCR). In the follow-up cohort including 102 patients, blood samples (plasma) were collected before the treatment initiation, 3 months, 6 months, and 12 months after treatments, and at the time of any recurrence or metastasis. Among these plasma miRNAs, miR-9-3p, miR-124-3p, miR-892b, and miR-3676-3p were significantly upregulated (*P* = 0.018, *P* = 0.039, *P* = 0.001, and *P* = 0.01, resp.) after treatment compared with pretreatment, and the four plasma miRNAs were downregulated again at recurrence or metastasis (*P* < 0.001, *P* = 0.015, *P* = 0.003, and *P* = 0.026, resp.). The dynamic changes in plasma miRNAs after treatment reflect the outcome of the disease and have the potential to monitor recurrence and metastasis in patients with NPC.

## 1. Introduction

Nasopharyngeal carcinoma (NPC) is one of the common malignant tumors in Southeast Asia with a high rate of local invasion and locoregional lymphatic metastasis [1]. Currently, the incidence of NPC in Southern China is especially high, at nearly 30 per 100,000 of population [2]. Despite the great advances in intensity-modulated radiotherapy, more than 30% of patients eventually develop recurrence or distant metastasis. Recurrence or metastasis is still the leading cause of death when curative treatment is no longer possible [3]. The comprehensive assessment of tumor response includes clinical examination, nasendoscopy with or without biopsy, Epstein–Barr virus (EBV) DNA or virus capsid antigen-specific IgA (VCA-IgA) titer measurement, and radiological imaging [4]. EBV is important in the development and progression of NPC. The plasma EBV-DNA level is currently recognized as the prognostic factor for NPC, but fake positive and negative results may be obtained due to the universality of EBV infection [5]. Recent studies have shown that specific genetic changes, such as dysregulation of microRNA expression or DNA methylation, may also predict the diagnosis and prognosis of NPC [6, 7]. Nevertheless, several issues need to be overcome before clinical genetic testing can become routine, including discrepancies between individual cases and inconsistencies in detection standards. This leads to a pressing need to identify an efficient tool to monitor recurrence or metastasis during the follow-up period of NPC, especially in Southern China.

MicroRNAs (miRNAs) are small noncoding RNAs that play an important regulatory role in a wide range of biological and pathological processes, especially in tumorigenesis and progression of various cancers [8]. Circulating miRNAs (plasma/serum miRNAs) have been proved to be present in the bloodstream in a remarkably stable form and serve as promising novel minimally invasive biomarkers for diagnosis and prognosis in multiple cancers, such as lymphoma and breast, prostate, ovarian, pancreatic, gastric, colorectal, and lung cancer [9–11]. However, identifying tumor-specific miRNAs is hampered by the fact that blood also contains miRNAs derived from other physiological or pathological activities, whose levels correlate with individual differences [12]. Additionally, the levels of circulating miRNAs generally fluctuate between different patients, and it is difficult to define a normal range of circulating miRNA levels. Therefore, some researchers believe that a paired comparative study between pre- and posttreatment can provide more accurate results to evaluate the therapeutic outcome and monitor tumor recurrence or metastasis [13, 14].

Several reports are available about circulating miRNAs in NPC tumorigenesis [15]. Liu et al. reported that the plasma five-miRNA-based biomarker model might provide a novel strategy for NPC diagnosis and prognosis [16]. Moreover, the four-serum miRNA signature might add prognostic value to the TNM staging system and provide information for personalized therapy in NPC [17]. Zhang et al. indicated that circulating EBV miRNAs might constitute new useful serological biomarkers for diagnosing NPC and predicting treatment efficacy [18]. However, the dynamic changes in these dysregulated miRNAs at different time points using self-paired plasma samples during the regular follow-up have never been explored.

In a previous study, the microarray analysis was performed to identify the miRNA expression signature in patients with NPC, and 33 differentially expressed miRNAs were identified. Among these 33 miRNAs, the low level of plasma miR-9 was significantly correlated with worse lymphatic invasion and advanced TNM stage. Interestingly, the plasma miR-9 was significantly elevated in 3-month posttreatment plasma compared with that in pretreatment samples [19]. This study aimed to analyze the dynamic changes in these dysregulated miRNAs in self-paired plasma samples after radiochemotherapy and explore their clinical potential as useful noninvasive biomarkers for monitoring recurrence and metastasis in patients with NPC.

## 2. Materials and Methods

### 2.1. Patient Cohort

A total of 122 patients newly diagnosed with NPC and 45 healthy donors were recruited prospectively from Nanfang Hospital (Southern Medical University, Guangzhou, China) between January 2011 and July 2014. None of them had received radiotherapy or chemotherapy earlier. Before treatment, blood samples taken from 20 patients and 10 healthy donors were used for miRNA microarray analysis, and additional samples from 102 patients and 35 healthy donors were used for validation. Then the 102 patients were followed up to September 2016 after treatment; those who could not return on time were excluded. The healthy donors were carefully selected to match the gender and age distribution of patients with NPC. The clinical classification for NPC was based on the seventh edition of the Union for International Cancer Control (UICC) TNM staging system. All patients were treated with a uniform protocol of image-guided intensity-modulated radiotherapy and/or cisplatin-based concurrent chemotherapy following induction chemotherapy according to the National Comprehensive Cancer Network Guidelines [20]. Informed consent was obtained from all individuals, and the research protocols were approved by the ethics committee of Nanfang Hospital.

After the completion of all treatments, the patients with NPC were followed up for 12–36 months, once every 3 months during the first and second years and every 6 months thereafter. All tumor recurrences and metastases were documented by imaging studies along with pathological verification if the lesions were accessible and the patient agreed. In the long-term follow-up study, the blood samples of patients with NPC were collected at four time points, including before treatment and 3 months, 6 months, and 12 months after treatment. Once tumor recurrence or metastases occurred, the blood samples were collected timely before the salvage therapy was performed.

### 2.2. Plasma Sample Collection, RNA Isolation, and miRNA Microarray Analysis

Whole blood (8 mL) was drawn into ethylene diamine tetraacetic acid- (EDTA-) containing tubes, mixed well, and kept at room temperature. Then, blood samples were centrifuged at 1500*g* for 10 min to separate cellular fractions and plasma. Finally, aliquot plasma was stored at −80°C until further processing. All blood samples were processed and completed within 4 h after collection.

Total RNA was extracted from plasma specimen with the TRIzol LS reagent (Invitrogen, CA, USA) and the RNeasy Mini kit (Qiagen, CA, USA) according to the manufacturers' instructions. In detail, 500 mL of plasma was mixed thoroughly with 1.5 mL of TRIzol LS reagent, incubated for 5 min at room temperature, and subsequently mixed with 400 mL of chloroform. The aqueous phase containing RNA was carefully removed, and RNA was precipitated by adding 100% ethanol. The mixture was applied to an RNeasy Mini spin column and washed several times. RNA was eluted and stored at −80°C until further processing.

Small RNAs extracted from the plasma samples were labeled using the miRCURY Hy3/Hy5 Power labeling kit and hybridized on the miRCURY LNA Array (Version 16.0, Exiqon, Vedbaek, Denmark). The data was analyzed using GeneSpring GX Software (version 9.0, Agilent Technologies, CA, USA). The threshold value for differentially expressed miRNAs was a fold change > 1.5 with a* P* value < 0.05. The candidate miRNAs were further filtered on the basis of expression levels.

### 2.3. miRNA Quantitative Real-Time Polymerase Chain Reaction

Quantitative reverse transcription-polymerase chain reaction (PCR) was performed for miRNA quantification using All-in-One miRNA First-Strand cDNA Synthesis Kit and All-in-One miRNA qPCR Kit (GeneCopoeia, MD, USA). The probes were purchased from Invitrogen (Invitrogen, CA, USA). The sequences are listed in Table 1. All reactions were run in triplicate on the Stratagene Mx3005p Real-Time qPCR System (Agilent Technologies, CA, USA). The average levels of U6 small nuclear RNA and two miRNAs (miR-634 and miR-1228-3p), which were not differentially expressed in the microRNA microarray, were used as internal control in plasma miRNA analysis. The differential expression level of plasma miRNA was calculated by the following equation:

(1) ΔCtmiR=CtmiR−CtmiR-U6+CtmiR-634+CtmiR-12283ΔΔCt=ΔCttest−ΔCtcontrol.

Fold changes were calculated through relative quantification: 2^−ΔΔCt^ [21].

### 2.4. Statistical Analysis

SPSS 16.0 software was used for statistical analysis (SPSS, IL, USA). The Mann–Whitney *U* test was performed to detect differences in characteristics of study participants and identify differentially expressed miRNAs between NPC and control groups. The expression levels of miRNAs before and after treatment were compared using the paired *t*-test. Repeated-measure analysis of variance (ANOVA) was used for plasma miRNA levels before and 3, 6, and 12 months after treatment, with the least significant difference tests for multiple comparisons. A *P* value less than 0.05 was considered statistically significant.

## 3. Results

### 3.1. Patient Characteristics

The characteristics of the study participants are presented in Table 2. No significant difference was found in the distribution of age and gender between the two cohorts. During the follow-up of 102 patients with NPC, 12 cases had recurrence or metastasis at different time points. The time and sites of recurrence or metastasis are listed in Table 3.

### 3.2. Selection and Validation of NPC-Associated Plasma miRNAs

**Figure 1:**
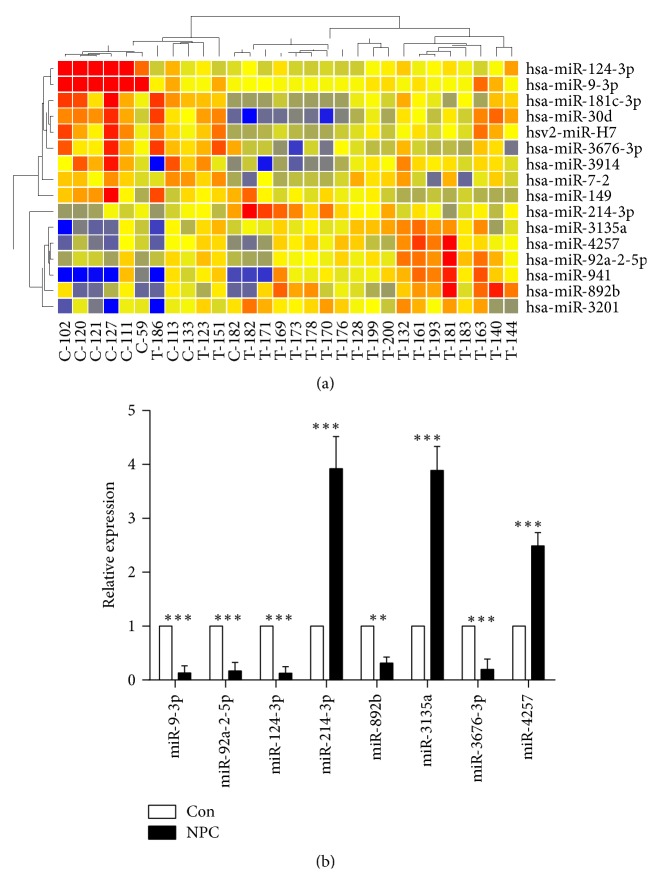
Selection and validation of plasma miRNA in NPC patients. (a) Heat map of differentially expressed microRNAs from GeneSpring software between the NPC group (*n* = 20) and the healthy control group (*n* = 10). Sixteen miRNAs were identified (*P* < 0.05). A vertical branch showed the expression pattern of candidate miRNAs in each individual. The relative expression was depicted according to the color scale shown in the figure. Red indicates upregulation and blue downregulation. Numbers with T indicate patients with NPC; numbers with C indicate healthy controls. (b) Validation of dysregulated miRNAs by qRT-PCR. An independent validation cohort included 102 patients with NPC and 35 healthy controls. Quantification was presented as mean values (error bars corresponded to standard deviation) relative to control from three independent experiments. ^*∗∗*^*P* < 0.01 and ^*∗∗∗*^*P* < 0.001.

Based on microarray analysis, 16 miRNAs were screened by the two-dimensional hierarchical clustering analysis, which showed significantly altered levels (Figure 1(a)). Among these 16 miRNAs, the levels of eight differentially expressed miRNAs were tested using an independent cohort (including 102 patients with NPC and 35 healthy volunteers) with quantitative reverse transcription- (qRT-) PCR to confirm the dysregulation of candidate miRNAs. The relative level of each miRNA is shown in Figure 1(b). The results demonstrated that five miRNAs (miR-9-3p, miR-92a-2-5p, miR-124-3p, miR-892b, and miR-3676-3p) were downregulated and three miRNAs (miR-214-3p, miR-3135a, and miR-4257) were upregulated in the plasma of patients with NPC.

### 3.3. Dynamic Changes in Plasma miRNA Levels after Treatment

**Figure 2:**
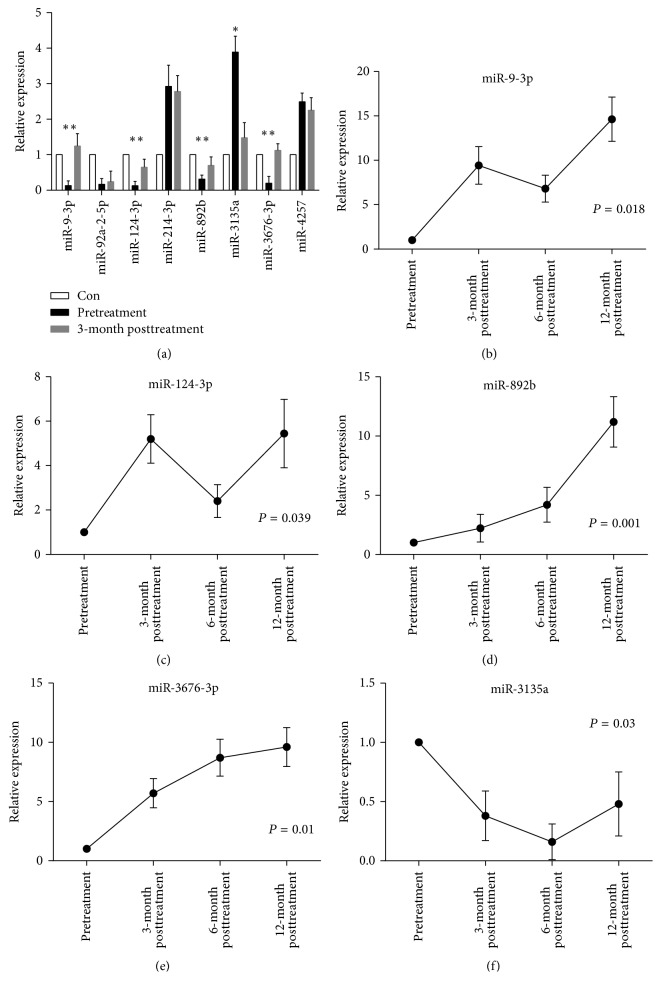
Dynamic changes in plasma miRNAs levels between pre- and posttreatment. (a) Levels of eight plasma miRNAs were examined in the paired samples of pretreatment and 3-month posttreatment of 90 patients compared with 35 healthy controls. The levels of five plasma miRNAs (miR-9-3p, miR-124-3p, miR-892b, miR-3135a, and miR-3676-3p) were significantly dysregulated and showed a tendency to go back to basic normal levels at 3-month posttreatment. (b–f) Dynamic changes in five plasma miRNA levels at three time points after treatment. Repeated-measure analysis of variance (ANOVA) was used to analyze the differences before treatment and 3, 6, and 12 months after treatment. ^*∗*^*P* < 0.05; ^*∗∗*^*P* < 0.01.

The 102 patients with NPC were followed up on time. The paired plasma samples of these patients were collected at different time points, including before, 3 months, 6 months, and 12 months after treatment. The 12 patients with recurrence or metastasis were excluded. The dynamic changes in 8 plasma miRNAs of 90 patients were analyzed during the follow-up period, and the 35 healthy donors were used as controls. As shown in Figure 2(a), the levels of 4 plasma miRNAs (miR-9-3p, miR-124-3p, miR-892b, and miR-3676-3p) were elevated at 3-month posttreatment compared with pretreatment. In contrast, the level of miR-3135a was reduced after treatment. The expression of these five differentially expressed plasma miRNAs tended to go back to basic normal levels after treatment compared with that before treatment. However, the expression levels of another three miRNAs (miR-92a-2-5p, miR-214-3p, and miR-4257) showed no significant differences between pre- and posttreatment. The levels of plasma miR-9-3p and miR-124-3p were significantly elevated at 3-month posttreatment, slightly reduced at 6-month posttreatment, and then continued to be elevated at 12-month posttreatment (Figures 2(b) and 2(c)). The levels of plasma miR-892b and miR-3676-3p showed a gradual increase during the follow-up (Figures 2(d) and 2(e)), while the level of miR-3135a showed a gradual decrease over 1 year after treatment (Figure 2(f)). In summary, the dynamic alterations of plasma miRNA levels were observed in patients with NPC after radiochemotherapy during the long-term follow-up.

### 3.4. Different Changes in Plasma miRNAs Levels at the Time of Recurrence or Metastasis

**Figure 3:**
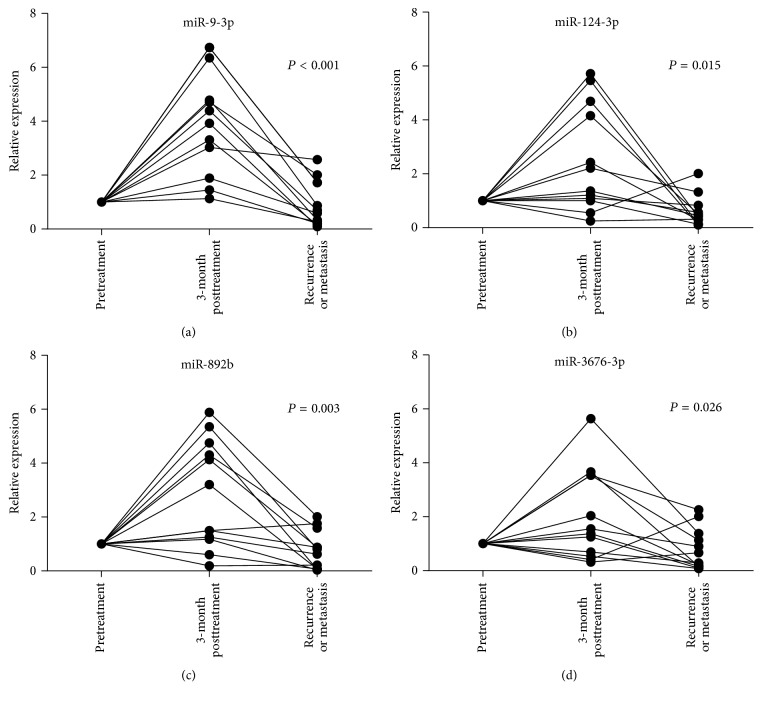
Dynamic changes in plasma miRNAs levels in NPC cases at the time of recurrence or metastasis. The levels of plasma miR-9-3p, miR-124-3p, miR-892b, and miR-3676-3p were statistically significantly upregulated at 3-month posttreatment and downregulated at recurrence or metastasis compared with pretreatment. Each individual value was represented by a circle, and the two points corresponding to the same individual were connected by a line.

Most recurrences and metastases (8/12, 66.7%) occurred within 1 year. Liver metastasis (5/12, 41.7%) and local recurrence (4/12, 33.3%) were more common. The levels of the eight differentially expressed plasma miRNAs were detected before the salvage treatment. As shown in Figure 3, the levels of four plasma miRNAs (miR-9-3p, miR-124-3p, miR-892b, and miR-3676-3p) statistically were elevated at 3-month posttreatment and decreased at recurrence or metastasis compared with pretreatment (*P* < 0.05). However, the levels of these 4 miRNAs kept the recovery trend to basic normal levels in another 90 cases of the follow-up cohort without recurrence or metastasis (data not shown). These data suggested that the dynamic changes in these four miRNAs had a potential value as biomarkers for monitoring recurrence and metastasis in patients with NPC.

## 4. Discussion

The aim of this study was to identify dynamic changes in differentially expressed plasma miRNAs in the posttreatment of patients with NPC, especially at the time of recurrence or metastasis, and investigate the potential of plasma miRNA as a biomarker for predicting recurrence or metastasis in patients with NPC. Based on systematic microarray analysis and RT-qPCR validation, the significantly declined level of four plasma miRNAs (miR-9-3p, miR-124-3p, miR-892b, and miR-3676-3p) in patients with NPC showed recovery trend to basic normal levels after treatment but declined again at the time of recurrence or metastasis. These results suggested the potential of the miRNAs as biomarkers for monitoring recurrence and metastasis in patients with NPC.

Circulating miRNAs have been identified as potential noninvasive biomarkers for diagnosis and prognosis in multiple cancers [22]. Several studies showed tumor-specific changes in circulating miRNAs in patients with NPC. Zeng et al. found that four serum miRNAs, including miR-17, miR-20a, miR-29c, and miR-223, were differentially expressed in the serum of patients with NPC and proposed that these miRNAs might serve as potential biomarkers for NPC diagnosis [15]. Zhang et al. reported that EBV-encoded miRNAs, miR-BART7 and miR-BART13, might constitute useful new prognostic serological biomarkers for predicting treatment efficacy of NPC [18]. Another study found that differentially expressed plasma miRNAs, as identified by next-generation sequencing, could be helpful in predicting survival in patients with NPC [17]. All these studies suggested that circulating miRNA might serve as new biomarkers for NPC. However, the dynamic changes in these dysregulated miRNAs at different time points using self-paired plasma samples during the regular follow-up have never been explored.

Our previous study found that the level of plasma miR-9 could distinguish locoregional from metastatic NPC cases with high sensitivity and specificity and was associated with clinical stages of NPC [19]. In this study, these eight plasma miRNAs screened from microarray were further identified by RT-qPCR in another follow-up cohort. All these eight plasma miRNAs were found to be differentially expressed between advanced stage and early stage of patients with NPC. The reason for this phenomenon was still not clear. Kosaka et al. suggested a secretary machinery of circulating miRNAs and their intercellular transfer and supposed that these circulating miRNAs might function as a pathway signal [22]. Further analysis is needed to clarify the origin of extracellular circulating miRNAs.

The diversification of circulating miRNA origin strongly suggests the existence of considerable interindividual differences in miRNA expression. Leidinger et al. monitored the plasma miRNA expression pattern in patients with lung cancer over time after surgery and found that the differences in the overall miRNA patterns at different time points were significantly smaller than the differences between patients [23]. Paired plasma samples of pretreatment and posttreatment were collected in this study to eliminate the effects of interindividual differences on the results. Based on the results of microarray screening and qPCR validation, the study focused on 8 significantly dysregulated plasma miRNAs in the follow-up cohort including 102 patients with NPC and found that 5 plasma miRNAs (miR-9-3p, miR-124-3p, miR-892b, miR-3135a, and miR-3676-3p) were significantly differentially expressed between pre- and posttreatment. Interestingly, the results showed that the levels of these five miRNAs tended to go back to basic normal levels after treatment compared with that before treatment, suggesting their potential values in monitoring recurrence and metastasis in patients with NPC.

Recent studies identified circulating miRNAs as predictors of response to therapeutics, such as radiotherapy and anticancer agents [24]. The abundance of circulating miRNAs was reported to change in patients with head and neck squamous cell carcinoma after radiochemotherapy [25]. Therefore, the dynamic changes in five plasma miRNA levels were further detected in the follow-up cohort. As shown in Figure 3, the levels of five plasma miRNAs tended to go back to basic normal levels after radiochemotherapy compared with pretreatment. The results suggested that these five miRNAs might have potential values in evaluating the therapeutic outcome of patients with NPC.

Circulating miRNAs have already been reported to predict the risk of recurrence in multiple cancers, including thyroid cancer [26], colon cancer [27], prostate cancer [28], and adrenocortical cancer [29]. In the long-term follow-up period, 12 cases had recurrence or metastasis at different time points after treatment. Also, the blood samples were collected, and the levels of the aforementioned five miRNAs were examined when the patient developed recurrence or metastasis. Surprisingly, the levels of four plasma miRNAs (miR-9-3p, miR-124-3p, miR-892b, and miR-3676-3p) were statistically significantly reduced at recurrence or metastasis. These results suggested that these four plasma miRNAs might serve as predictive biomarkers for monitoring recurrence and metastasis in patients with NPC. Although quantitative real-time PCR has been widely used to examine the expression of circulating miRNAs, still many arguments exist regarding the choice of the internal control. U6 and miR-16 were the most frequently used controls in recent studies on circulating miRNAs. However, U6 was reported to be absent in serum or plasma unless cell lysis occurred, and differential cell lysis was possible in diseases [30]. Additionally, hemolysis may cause up to 50-fold alteration of circulating miRNA levels [31]. In the present study, the dysregulated level of miR-16 was observed in the plasma of patients with NPC compared with healthy controls, whereas the levels of miR-634 and miR-1228 were relatively stable in miRNA microarray profiling. Recently, miR-1228 was also proved to be a stable endogenous control for quantifying circulating microRNAs in cancer patients [32]. Therefore, the average level of U6, miR-634, and miR-1228 was used as an internal control.

Recent studies showed that the levels of circulating miR-9 and miR-124 were dysregulated, which might act as diagnostic and prognostic biomarkers in multiple cancers, including lung cancer, esophageal squamous cell carcinoma, pancreatic ductal adenocarcinoma, and prostate cancer [33–36]. In NPC, our previous studies showed that both miR-9 and miR-124 functioned as tumor suppressors by targeting chemokine receptor-4 (CXCR4) and forkhead box Q1 (FOXQ1), respectively [37, 38]. It has also been reported that miR-9-3p suppresses the epithelial-mesenchymal transition (EMT) in NPC cells by downregulating fibronectin 1 (FN1), *β*1 integrin (ITGB1), and *α*5 integrin (ITGAV) [39], while miR-124 suppresses tumor growth by targeting calpain small subunit 1 (CAPN4) and signal transducer and activator of transcription 3 (STAT3) [40, 41]. Although it has been reported that miR-892b regulates the p19ARF/cyclin D1/CDK6 and Sp-1/MMP-9 signaling networks in bladder cancer and miR-3676 regulates T-cell leukemia/lymphoma 1 (TCL1) in chronic lymphocytic leukemia [42, 43], the role of the three miRNAs (miR-892b, miR-3135a, and miR-3676-3p) has never been reported in NPC. Further investigations are needed to reveal their functions and mechanisms in NPC tumorigenesis. Some of the NPC-related miRNAs found in previous studies were not identified in this study, including targeting of STAT3 by miRNA-98, targeting of TGF*β*R2 by miR-93, and targeting of metastasis-associated gene 2 by miRNA-148b [44–46]. These partially inconsistent results may reflect differences in sample types, screening tools, or quantification methods. EBV-DNA and VCA-IgA have been tested routinely in NPC patients, because EBV infection is associated with the development of NPC. Positive EBV-DNA or VCA-IgA test might be a prognostic factor for the relapse and survival of NPC patients, but these markers do not always appear to be reliable [47].

We recognize that our study has limitations, including the fact that it was an observational study with limited sample size, and that the sensitivity and specificity of the miRNA biomarkers should be compared with conventional NPC markers such as EBV-DNA and VCA-IgA. Further prospective clinical trials with larger sample sizes, multicenter studies, and long-term follow-up should be performed to verify the specificity and sensitivity of these miRNA biomarkers.

In summary, this study clearly demonstrated that the dynamic changes in plasma miRNAs could be useful blood-based biomarkers for predicting the therapeutic response of patients with NPC. These noninvasive blood-based biomarkers might have great potential in predicting the therapeutic response after radiochemotherapy and monitoring recurrence or metastasis. Therefore, our findings may suggest a new approach for the clinical follow-up of patients with NPC.

## Figures and Tables

**Table 1:** All detected plasma miRNA and upstream primer sequences.

miRNA	Sequence (5′-3′)	Primer (5′-3′)
hsa-miR-9-3p	AUAAAGCUAGAUAACCGAAAGU	ATAAAGCTAGATAACCGAAAGT
hsa-miR-92a-2-5p	GGGUGGGGAUUUGUUGCAUUAC	GGGTGGGGATTTGTTGCATTAC
hsa-miR-124-3p	UAAGGCACGCGGUGAAUGCC	TAAGGCACGCGGTGAATGCC
hsa-miR-214-3p	ACAGCAGGCACAGACAGGCAGU	ACAGCAGGCACAGACAGGCAGT
hsa-miR-892b	CACUGGCUCCUUUCUGGGUAGA	CACTGGCTCCTTTCTGGGTAGA
hsa-miR-3135a	UGCCUAGGCUGAGACUGCAGUG	TGCCTAGGCTGAGACTGCAGTG
hsa-miR-3676-3p	CCGUGUUUCCCCCACGCUUU	CCGTGTTTCCCCCACGCTTT
hsa-miR-4257	CCAGAGGUGGGGACUGAG	CCAGAGGTGGGGACTGAG
U6	CGCAAGGAUGACACGCAAAUUCGU	CGCAAGGATGACACGCAAATTCGT
hsa-miR-634	AACCAGCACCCCAACUUUGGAC	AACCAGCACCCCAACTTTGGAC
hsa-miR-1228-3p	UCACACCUGCCUCGCCCCCC	TCACACCTGCCTCGCCCCCC

**Table 2:** Patient and disease characteristics.

Characteristics	Follow-up cohort (*n* = 102)	Healthy control (*n* = 35)	*P*
Age (year)	46.08 ± 9.76	43.15 ± 8.42	0.748
Gender, *n* (%)			0.965
Male	76 (74.5)	26 (74.3)	
Female	26 (25.5)	9 (25.7)	
T stage, *n* (%)			
T1	6 (5.9)		
T2	28 (27.4)		
T3	38 (37.3)		
T4	30 (29.4)		
N stage, *n* (%)			
N0	16 (15.7)		
N1	32 (31.4)		
N2	43 (42.1)		
N3	11 (10.8)		
M stage, *n* (%)			
M0	99 (97.1)		
M1	3 (2.9)		
UICC stage, *n* (%)			
I	1 (0.9)		
II	22 (21.6)		
III	43 (42.2)		
IV	36 (35.3)		

**Table 3:** Characteristics of recurrence or metastasis cases.

ID	Gender	Age (year)	TNM stage	Time (month)	Recurrence or metastasis sites
Case 1	Male	42	T3N1M0	20	Recurrence
Case 2	Male	39	T3N2M0	8	Lung
Case 3	Male	29	T4N3M0	12	Liver
Case 4	Female	40	T3N1M0	24	Bone
Case 5	Male	45	T1N3M0	24	Cervical lymph node
Case 6	Male	30	T2N3M0	6	Liver
Case 7	Female	50	T4N1M0	6	Recurrence
Case 8	Female	48	T2N3M0	6	Liver
Case 9	Male	56	T3N2M0	12	Liver
Case 10	Male	58	T4N2M0	9	Recurrence
Case 11	Male	33	T4N3M0	30	Liver
Case 12	Male	48	T4N2M0	10	Recurrence
